# Do psychiatry and neurology need a close partnership or a merger?^†^

**DOI:** 10.1192/pb.bp.113.046227

**Published:** 2015-06

**Authors:** Michael Fitzgerald

**Affiliations:** 1 Trinity College Dublin, Ireland

## Abstract

Advances in neuroscience in recent years have blurred the boundaries between psychiatry and neurology. They now have more in common than what divides them and this signals a return to their origins. Many have called for a merger of the two disciplines, which would offer a more holistic approach, whereas others vigorously reject such a move. Limiting neurology to the study of the nervous system and psychiatry to the social brain or affect and its disorders is no longer sustainable. The ongoing separation of the disciplines has had an impact on diagnosis and treatment, on professional isolation and on funding psychiatric research.

The relationship between psychiatry and neurology remains a controversial topic, with strong voices opposing a merger, whereas others point out that the future lies with the neurologist/psychiatrist or neuropsychiatrist.^1^ Either way, it highlights the growing disparity in how the disciplines are currently defined and the many areas of overlap.

## History of the relationship between neurology and psychiatry

The term ‘neurology’ originated with English physician Thomas Willis following his study of brain anatomy in the 1660s, according to Millon.^2^ Subsequently in 1808, Johann Christian Reil, a German physician and philosopher, gave us the term ‘psychiatrie’. However, the two disciplines had common origins. Krishnamoorthy^3^ points out that the ancients, including Hippocrates, believed that all psychopathology arose in the brain and that this is clearly reflected in writings well into the nineteenth century. On the other hand, this was not a universally held view and some of the ancients believed that hysteria was because of a wandering womb. Even so, the 1845 seminal work on mental illness by Wilhelm Griesinger in *Mental Pathology and Therapeutics* still has a modern ‘ring’ to it in the 21st century. He was a major clinical and academic researcher in a number of hospitals in Europe at the time. The one-time unity of the disciplines was also reflected in the title of the journal Griesinger founded in 1867, *Archives of Psychiatry and Neurology*. For Griesinger, mental diseases were essentially brain diseases, according to Millon.

Over the years there has been considerable opposition to Griesinger. In an interview with Poole,^4^ William Alywn Lishman was severely critical of Griesinger because he ‘tried to make the whole of psychiatry brain science’. I would concur with Lishman that Griesinger went too far and especially ignored environmental influences. I believe the biopsychosocial model of psychiatry can only operate via the brain and Johansson^5^ is correct in his assertion that all mental life will eventually be mapped onto ‘a neuronal substrate’. The split between medicine and psychiatry was lamented by Silas Weir Mitchell as early as 1894. He expressed the view that when the ‘treatment of the insane’ passed completely out of the hands of the profession at large and ‘into those group of physicians (psychiatrists) who constitute a sect apart... what evil this has wrought, what harm it has done to us’, as noted by Andreason.^6^ This split became even more pronounced in the USA between 1935 and 1975, when psychoanalysis largely took over psychiatry there, further increasing the gap between neurology and medicine generally. Ironically, psychoanalysts are now trying to reconnect with neurology through neuropsychoanalysis, according to Solms.^7^

The advent of diagnostic imaging has brought a certain degree of clarity to the field. Kandel points out that had imaging been available in 1895 when Freud wrote *Project for a Scientific Psychology* he might have directed psychoanalysis along very different lines, keeping it ‘in close relationship with biology as outlined in that essay’.^8^ It is worth noting that Freud was a superb neuropathologist and neurological scientist, as evidenced in the very ambitious but highly relevant *Project for a Scientific Psychology*.^9^ However, the work also signalled his subsequent move away from traditional science.

## Why are psychiatry and neurology separate?

Many views have been expressed on why psychiatry and neurology are separate. In response to White e*t al*’s^10^ assertion that it is time to end the distinction between mental and neurological illnesses, Ikkos points out that neurology’s expertise is the nervous system and its disorders, whereas psychiatry’s expertise is affect and its disorders.^11^ Furthermore, he argues that, conceptually, neurology is necessarily at a different level of abstraction from the nervous system. This argument, however, is not the least bit persuasive to me. Miller notes that ‘psychiatry was neurology without physical signs’,^12^ whereas Holmes points out that ‘only psychiatry can encompass the social brain’.^13^ There is absolutely no reason why neurologists cannot embrace the social brain and indeed many do today. I believe the split between neurology and psychiatry is in fact artificial.

The chorus of disapproval against neuropsychiatry has certainly grown. Pies argues that psychiatry and neurology cannot simply merge because they use ‘significantly different narratives or... discourses’.^14^ He also claims that psychiatry is grounded in human subjectivity and existential concerns and is a ‘discourse of interlacing and multi-layered meanings’ and a ‘narrative about narratives’. Neurology, on the other hand, he believes is fundamentally ‘a discourse of neuroanatomical and neurophysiological relationships’. When a neurologist examines a patient with symptoms not corresponding to known neurological pathways, which Pies sees as ‘functional, supratentorial or psychogenic’, then the patient should be deferred or referred to the psychiatrist. Essentially, Pies’ description of psychiatry has echoes of psychoanalysis. For example, he asserts that psychiatry’s discourses should be understood as a dialectic between a text and a presumed subtext – ‘not unlike the dialectic between *p’shat* and *d’rash* in Talmudic exegesis. That is, beneath the literal words or surface meaning of a biblical text (*p’shat*), there lies a realm of figurative, allegorical, and mystical meanings that must be explicated (*d’rash*)’.^14^ Indeed, his description of psychiatry relates to that of the mid-nineteenth century, in my view.

An editorial by Baker *et al* in the *BMJ* in 2012 stated that it is time to tear down the wall between neurology and psychiatry advances in neuroscience.^15^ In response to this editorial, Bailey *et al* presented the orthodox psychiatric reaction. They point out that most mental disorders, given our current state of knowledge, have no unequivocal biomarkers and classification has to rely, however imperfectly, on clinical signs and symptoms.^16^ They were against a merger of neurology and psychiatry, but in favour of a close working relationship.

The advances in neuroscience prompted Stone & Sharpe to pose a key question: will greater understanding of neuroscience mean that psychiatry will simply follow neurology in abandoning the patients that fail to fit into a reductionistic paradigm?^17^ However, I believe that if there were a merger, patients would be less likely to fall through the ‘cracks’. Excessive specialisation is the greatest reason for bringing neurology and psychiatry together. Indeed, even at a research level progress is more likely to occur at the interfaces between specialties and subspecialties. One reason for the lack of progress in psychiatric research in recent years has been because of the excessive specialisation and subspecialisation.

## Why bring neurology and psychiatry together?

One of the most compelling arguments for bringing the two disciplines together is that their boundaries are becoming increasingly blurred. Ramachandran observed this fact and declared it was only a matter of time before psychiatry becomes just another branch of neurology.^18^ I would dispute that aspect of the argument; there is no question of one discipline ‘swallowing’ up the other. Instead it would be a merger of two equal partners: neurology and psychiatry. If it were to occur, both disciplines would enrich each other enormously.

The separation of the two disciplines has had a somewhat negative impact on diagnosis and treatment. Kanner points out that, in neurology, the separation from psychiatry has led to comorbid disorders being underrecognised and undertreated.^19^ In effect, the separation of neurology from psychiatry has led to a separation of the brain from the mind – the physical from the mental – which has been unhelpful for both disciplines. If a merger did occur, the neuropsychiatrist could provide a more holistic approach to the diagnosis and treatment of a patient. In fact, all neurologists and psychiatrists practise basic counselling and brief therapy to varying degrees. It is noteworthy that there are similar brain changes after the treatment of obsessive-compulsive disorder with either medication or behaviour therapy. This increases the link somewhat between neurology and psychiatry.

Aarli points out that psychiatry and neurology have a common route and both share a common basis in neuroscience.^20^ He also notes that there is much more that unites neurology and psychiatry than divides them. Neurobiological conditions like epilepsy, autism, dementia, delirium, Tourette syndrome, intellectual disability, dyspraxia, speech and language problems are all overlapping. Between neurology and psychiatry Henningsen favours overcoming ‘dualistic’ and often ‘irrational splits’ in the classification and in the practice of medicine.^21^ He agrees with the idea of subsuming mental disorders under ‘disorders of the brain’ because this gives greater clarity and simplicity. Kandel finds it useful to consider that psychiatry and psychoanalysis work at the level of individual nerve cells and their synaptic connections.^8^ Neurology and psychiatry are simply two ‘sides of the same coin’. Certainly in the area of neural plasticity, neurology and psychiatry overlap.

The overlap is also evident in medical journals relevant to the disciplines. In a study of papers published in *Neurology* and the *American Journal of Psychiatry*, Price^22^ found that less than 5% of papers in the *American Journal of Psychiatry* were on meningitis, epilepsy and headache and that less than 5% of papers in *Neurology* focused on schizophrenia, panic and mania. The proportions for attention-deficit hyperactivity disorder were 23% in *Neurology* and 77% in the *American Journal of Psychiatry*; for autism 30% in *Neurology* and 70% in the *American Journal of Psychiatry*; for ‘mental retardation’ 70% in *Neurology* and 30% in the *American Journal of Psychiatry*. As one can see, there is considerable overlap. Similarly, Raja showed that neurological disease affected 13.05% of acute and 68.9% of chronic psychiatric patients.^23^

## Professional isolation from medicine

The question of professional isolation has also emerged. The separation of psychiatry from neurology has led Levine to comment that, over the past 30 years, psychiatry has become professionally, geographically and managerially separate from the rest of medicine.^24^ In many places this isolation has seriously damaged psychiatry and caused major recruitment and funding problems.

In a paper entitled ‘Wake-up Call for British Psychiatry’, Craddock *et al* were concerned about the evolution of unclear responsibility in psychiatry, which reduces medical student interest because of not being ‘proper doctors’, and modern psychiatry, diminishing the value of careful diagnosis and reducing psychiatry to a ‘nonspecific psychological support’.^25^ Combining neurology and psychiatry would reduce these problems. It is well-known that medicine and psychiatric illness are closely allied. The merger of neurologists and psychiatrists would improve the care of the patient at the interface and moreover may reduce stigma. Bullmore *et al*^26^ believe that the merger would reduce stigma, however Jorm & Oh^27^ did not find that brain *v.* social aetiology affected stigma in their formal study. Read *et al*,^28^ in their review paper, said that biological psychiatry increases stigma, whereas Bullmore *et al*^26^ suggested the opposite. This issue remains controversial and opinions as described vary.

There is a great deal of similarity in the training of neurologists and psychiatrists from medical school onwards. At the present time, all psychiatrists are required to spend a minimum of 6 months to a year working in neurology and vice versa. Joint training in neurology and psychiatry would be helpful. These individuals would be dual trained and would require both Royal Colleges to come together to produce this dual-trained neurologist/psychiatrist, as happens in the USA and Germany. Indeed, it may be easier to recruit this neurologist/psychiatrist in the future. In a study of trainers and trainees in psychiatry/neurology, Schon *et al*^29^ found that psychiatrists were even keener on links between neurology and psychiatry training than neurologists, with psychiatric specialist registrars significantly more in favour.

In conclusion, psychiatrists should return home to neurology and medicine and leave non-medical interventions to non-medical practitioners, for example in relation to specialist or long-term psychotherapy. Neurologists and psychiatrists need to merge into neuropsychiatry or some acceptable title. The merger would admittedly not be easy, but it would be beneficial to both fields in the long term and to patients at a clinical level.
